# A Case Report of Malaria Infection Following Laparoscopic Sleeve Gastrectomy

**DOI:** 10.7759/cureus.49683

**Published:** 2023-11-29

**Authors:** Mehmet Gençtürk, Nihal Sarıca Cırık, Muhammed Said Dalkılıç, Merih Yılmaz, Hasan Erdem

**Affiliations:** 1 Obesity Surgery, Dr. HE Obesity Clinic, Istanbul, TUR; 2 Microbiology, Istanbul Public Health Laboratory, Istanbul, TUR; 3 General Surgery, Marmara University, Istanbul, TUR; 4 General Surgery, Dr. HE Obesity Clinic, Istanbul, TUR

**Keywords:** plasmodium falciparum, trophozoite, malaria, fever, sleeve gastrectomy

## Abstract

A 41-year-old woman from the Democratic Republic of the Congo underwent laparoscopic sleeve gastrectomy (LSG) as a surgical treatment for obesity. Despite an unremarkable preoperative evaluation, the patient developed a fever and elevated C-reactive protein (CRP) levels postoperatively. Physical examination findings, laboratory tests, and imaging studies ruled out surgical complications, leading to the consideration of infectious causes. A thorough patient history revealed a residence in a malaria-endemic region with a history of recurrent malaria episodes. In addition to her complaints, the patient developed pancytopenia. The blood smear revealed the presence of ring forms of *Plasmodium falciparum* in red blood cells, along with other species of Plasmodium. The rapid diagnostic test (RDT) showed a positive result for the *P. falciparum* antigen, a negative result for the *P. vivax* antigen, and a positive result for the pan-antigen. Based on these findings, a mixed malaria infection was considered for the patient, and she was transferred to an advanced infectious disease hospital for specific typing and further treatment. The patient received prompt treatment and was discharged in stable condition. Malaria could potentially be among the uncommon factors leading to fever after bariatric surgery in patients from malaria-endemic countries. Surgical stress may exacerbate the course of a malaria infection.

## Introduction

Obesity has become a worldwide epidemic, with a substantial increase in obesity rates over the past 50 years [1]. Bariatric surgery stands out as the most effective long-term treatment for obesity and its associated comorbidities [2].

The surgical treatment of obesity has seen a rise in popularity, with laparoscopic sleeve gastrectomy (LSG) being the most commonly performed bariatric surgery globally [3]. LSG offers numerous advantages, including a shorter duration of the procedure and a faster recovery, while also proving to be effective for weight loss. This technique is characterized by its simplicity, involving only a restrictive component, which in turn reduces the risk of malnutrition [4].

In the current era, patients often opt to travel to foreign countries for surgical procedures like LSG for various reasons, including insufficient technical facilities in their home countries, a lack of experienced medical teams, high surgical costs, and the convenience of travel [5]. When obtaining the medical history of patients from different countries, it is crucial to consider local factors and any previous medical conditions that might significantly impact the treatment process.

Malaria is a protozoal systemic infection caused by unicellular parasites of Plasmodium. Untreated cases are associated with a high mortality rate. These parasites are transmitted to the human host through the bite of a female Anopheles mosquito. Malaria remains a substantial global threat, impacting half of the world's population and causing approximately 1 million deaths annually [6,7]. Characterized by its endemic nature and potential for recurrences, malaria presents a life-threatening challenge in terms of diagnosis and management, particularly for clinicians unfamiliar with the intricacies of the condition.

This study aimed to shed light on malaria infection, an exceedingly rare cause of postoperative symptoms characterized initially by fever and elevated C-reactive protein (CRP) following LSG.

## Case presentation

A 42-year-old woman from the Democratic Republic of the Congo struggled to achieve weight loss despite attempting various diets, lifestyle modifications, and engaging in physical activity for the past three years. Her body mass index (BMI) was 37.2 kg/m^2^, corresponding to a weight of 102 kg and a height of 166 cm. The preoperative medical history did not reveal any chronic diseases, prior surgeries, or a history of smoking or alcohol consumption.

After a comprehensive multidisciplinary evaluation, LSG was recommended as an appropriate treatment for obesity. Prior to the surgery, a preoperative evaluation was conducted, including a biochemical panel encompassing renal and hepatic functions, a complete blood count (CBC), a coagulation profile, thyroid function tests, a chest X-ray, an electrocardiogram, and abdominal ultrasonography. All preoperative test results were within the normal range, except for one finding: the preoperative CRP level was elevated at 21 (0-5) mg/L. Body temperature, pulse rate, blood pressure, and oxygen saturation were normal. No symptoms or clinical examination findings were identified to account for the elevated CRP levels. Following consultations, the anesthesiologist granted approval for the surgery.

Following the preoperative preparations, a standard LSG was performed without complications. The patient was extubated without any issues and monitored in the post-anesthesia care unit before being transferred to their wardroom. The patient began oral intake with water two hours later and was encouraged to start mobilizing at the fourth postoperative hour. Additionally, incentive spirometry respiratory exercises were performed at a rate of 20 times per hour. On postoperative day (POD) 1, the patient experienced a fever three times with a temperature of 38.5 °C, and her CRP level was elevated to 121 mg/L. She received intensified respiratory physiotherapy. She had two episodes of a fever reaching 38 °C, her CRP level increased to 188 mg/L, and her white blood cell count (WBC) decreased to 3340/mm^3^ on POD 2. An oral-intravenous contrast-enhanced computed tomography (CT) scan of the thorax and abdomen was conducted, but no abnormalities were detected. Urinalysis, urine, and blood cultures (during febrile episodes) were taken to investigate the possible source of infection. The urinalysis showed normal results. Empirical antibiotic treatment with ceftriaxone (1 g intravenous, twice a day) was initiated, and the patient's dietary regimen was adjusted to include 1.5 liters of water and 200 ml of milk containing 50 g of whey protein three times a day. On POD 3, her CRP level was 198 mg/L, and she did not have any fever. The urine culture yielded no signs of bacterial growth. However, she had an episode of fever at 38 °C on POD 4, and her CRP level rose to 209 mg/L. The blood culture showed no signal of bacterial growth. The biochemical panel, which assessed liver and kidney functions, yielded normal results. Oral intake was discontinued, and the patient's antibiotic therapy was switched to piperacillin-tazobactam (4.5 g intravenous, 3 times a day) on an empirical basis with a step-up approach. On POD 5, she remained without fever, but her CRP increased to 281 mg/L, and her CBC showed pancytopenia, with a WBC count of 2960/mm^3^ (4000-10,000), a platelet count of 59,000/mm^3^ (150,000-400,000), and a hemoglobin level of 9.6 g/dL (12-16) (Table 1). The ALT (alanine transaminase) level was 31 (4-36) units/L, the AST (aspartate aminotransferase) level was 30 (10-36) units/L, LDH (lactate dehydrogenase) was 348 (<247) units/L, GGT (gamma-glutamyl transferase) was 183 (<36) units/L, the total bilirubin was 1.54 (0.1-1.2) mg/dL, and the direct bilirubin was 0.48 (<0.3) mg/dL.

**Table 1:** Patient follow-up with key events, test results and vital signs.

	WBC (×10^3^/mm^3^)	CRP (g/L)	Hemoglobin (g/dL)	PLT (×10^3^/mm^3^)	Temperature (°C, max)	Pulse rate (per minute, min-max)	Key events
Pre-op	4.10	21	11.5	180	36.7	71–84	
POD 0	-	-	-	-	37	80–92	Uneventful surgery
POD 1	3.98	120	10.9	151	38.5 (3 times)	84–94	Incentive spirometer, watchful waiting
POD 2	3.34	188	10.9	125	38 (twice)	82–96	Thoracoabdominal CT: normal Urinalysis (normal), urine and blood cultures were obtained. Empiric ceftriaxone
POD 3	3.79	198	9.9	95	37	75–102	Urine culture: normal
POD 4	3.69	208	10.7	81	38.5 (once)	84–105	Blood culture: no signal empirical antibiotic upgrade to piperacillin-tazobactam
POD 5	2.96	281	9.6	59	37.2	94–106	Thorough patient history internal medicine consultation blood smear rapid diagnostic test referral and treatment

After taking a thorough patient history, the decision was made to refer the pancytopenia to the internal medicine department for further evaluation. Hepatosplenomegaly was not observed during the physical examination. The patient's history revealed a residence in a malaria-endemic African region, multiple previous malaria episodes, and a similar episode one year prior to surgery. These factors prompted the medical team to consider the possibility of a malaria infection.

**Figure 1:**
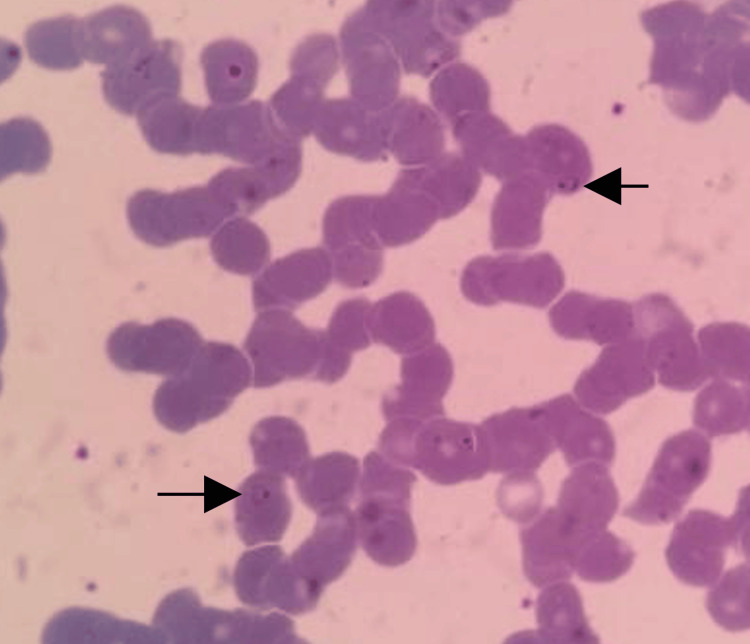
Microscopic image of peripheral thin blood smear. Arrows indicate ring-shaped trophozoites of plasmodium falciparum. Images of suspected non-falciparum plasmodium trophozoites, unfortunately, cannot be shown due to data loss.

A blood smear and a rapid diagnostic test (RDT) for malaria were conducted. The blood smear revealed the presence of ring forms of Plasmodium falciparum in red blood cells, along with other species of Plasmodium (Figure 1). The RDT showed a positive result for *P. falciparum* antigen, a negative result for *P. vivax* antigen, and a positive result for the pan-antigen. Based on these findings, a preliminary diagnosis of mixed malaria infection was considered for the patient.

The patient was transferred to an advanced infectious disease hospital for specific typing and further treatment, where she received immediate care. Six days after the initiation of malaria treatment, she was discharged with oral medications in a stable clinical condition, free of any symptoms. Following her return to her home country, monthly video consultations with her bariatric surgeon were arranged, during which her weight was monitored. During the following three months, she did not experience another episode of malaria and lost 20 kg during this period.

The patient gave full permission for the publication and other use of visual or textual material in the case. Informed consent and approval from the Institutional Review Board were obtained.

Technical details of the malaria diagnosis

Peripheral blood samples obtained from the patient underwent both microscopic examination and RDT to identify the presence of Plasmodium species. Thin smear and thick drop preparations stained with Giemsa were examined under a microscope with a ×100 immersion objective. Simultaneously, an analysis for the presence of Plasmodium species antigens was conducted using a malaria rapid diagnostic test (Standard Q Malaria P.f./Pan Ag., Malaria P.f./P.v. Ag., SD Biosensor, Inc., Suwon-si, Korea) following the manufacturer's instructions.

## Discussion

Diagnosing malaria in the postoperative period can be challenging due to various competing possibilities. This case report elucidated the diagnostic pathway for malaria in the postoperative period.

Fever is a common occurrence in the initial days following surgery and often presents a diagnostic and management challenge for the healthcare team. The threshold for defining fever can vary, with many considering 38 °C as the standard, but hospitals and units may use different thresholds [8]. It is crucial to distinguish between infectious and non-infectious causes of fever. Timing classifications (such as early, late, and delayed) may facilitate the differential diagnosis of postoperative fever. In the early postoperative period, fever is typically linked to non-infectious factors, while fever occurring after three days should primarily raise suspicion of an infectious origin [9].

Atelectasis is common postoperatively and has become established in classical textbooks and clinical practice as the most prevalent cause of early fever. Thus, incentive spirometry is widely employed in practice to address atelectasis. However, recent studies have emphasized that early fever occurs as a cytokine-related inflammatory response to surgical trauma and may complicate the cause-and-effect relationship because it is concurrent with atelectasis [10].

Fever in the early postoperative period, typically the first two to three days, sometimes referred to as "physiologic fever," usually resolves on its own and does not require further diagnostic investigation. However, assuming that postoperative fever is solely due to tissue damage and atelectasis can distract clinicians from exploring the actual underlying cause of the fever. Therefore, it is crucial to carefully review the patient's medical history and medications and conduct a thorough physical examination during this period [11].

CRP is an acute-phase reactant protein produced by the liver in response to inflammation, whether from infections or non-infectious inflammatory conditions. CRP levels tend to rise more rapidly and significantly in response to bacterial infections. However, any inflammatory condition has the potential to elevate CRP levels. Obesity is known to be an inflammatory condition, and as a result, individuals with obesity may have mildly elevated CRP levels even in the absence of noticeable symptoms [12,13]. Postoperative CRP monitoring is a common clinical practice used to diagnose and monitor infectious complications after major abdominal surgeries. When accompanied by fever, tachycardia, or relevant clinical examination findings, an elevated CRP level indicates a more clinically significant condition [14]. Also, CRP has been proposed as a predictive marker for the early detection of postoperative complications, including leakage following LSG. Some authors have even attempted to establish specific cut-off values based on postoperative timeframes [15,16].

In this case, the onset of fever and an elevated CRP level on POD 1 were initially attributed to atelectasis or a normal body response to surgery, as there were no accompanying clinical examination findings. Accordingly, a watchful-waiting approach was adopted. However, additional investigations were initiated due to the subsequent increase in CRP levels and persistent fever on POD 2. An empirical antibiotic was initiated. In such clinical presentations following LSG, the primary concern is to rule out staple line leakage and associated infectious complications. Consequently, a thoracoabdominal CT scan was conducted, which yielded normal results. Further investigations, including urinalysis and blood and urine cultures, were deemed necessary, but all returned normal. In light of the persistent fever and notable elevation in CRP levels, an empirical antibiotic step-up approach was implemented as a preventive measure against potential staple line leakage. Upon the recommendation of the internal medicine department, a thorough patient history was conducted, uncovering that the patient resided in a malaria-endemic region in Africa and had previously experienced multiple episodes of malaria. This comprehensive patient history, coupled with clinical observations and the presence of pancytopenia, led our medical team to consider the possibility of a malaria infection.

There are four main species of Plasmodium parasites that infect humans: *P. vivax*, *P. malaria*, *P. ovale*, and *P. falciparum*. *P. vivax* is the most prevalent species on a global scale, while the majority of fatal cases are attributed to *P. falciparum*. In individuals afflicted with malaria, the disease manifests as recurrent cycles of chills, shivering, fever, and sweating. Malaria suspicion necessitates immediate attention. Early diagnosis and the prompt initiation of effective treatment are fundamental cornerstones in the management of malaria cases [7].

The microscopic examination of the patient's peripheral blood sample is currently regarded as the "gold standard" for malaria diagnosis. This examination, known as a "thick drop and thin smear," identifies Plasmodium parasites. A thick blood smear is particularly useful for assessing the parasite load, while a thin blood smear is more effective in identifying the specific type of parasite [6]. Moreover, RDTs and polymerase chain reaction (PCR) tests capable of distinguishing Plasmodium species have been developed, and they offer a valuable alternative when reliable microscopic diagnosis is unavailable. RDTs detect antigens from malaria parasites and can provide results in less than 15 minutes. While RDTs are less sensitive than other laboratory tests, they serve as a practical and quick option for malaria diagnosis. PCR tests, although more sensitive than routine blood smear microscopy or RDTs, have a longer processing time, making them less suitable for quick initial diagnosis and treatment. However, they are essential for confirming the specific malaria parasite species and detecting mixed infections. This information is crucial for choosing the right antimalarial drugs to effectively treat the patient [7].

In this case, the patient was diagnosed with malaria, with a suspicion of a mixed infection, through a blood smear and RDT. Subsequently, the patient was referred to an advanced center for specific typing and further treatment. The onset of malaria symptoms shortly after surgery in this patient, who was asymptomatic before the procedure, complicates a definitive interpretation. It remains unclear whether the observed manifestation represents a relapse, reinfection, delayed clinical presentation of a silent parasitemia, or a combination of these factors, considering the available information.

Postoperative malaria cases suggest a potential role for surgical stress-induced immunosuppression [17,18]. The neuroendocrine response to surgery is recognized for its ability to suppress cell-mediated immunity. Several authors have proposed that factors impacting immunity, including infection or surgical stress, may contribute to malaria relapse [19]. Furthermore, a national study conducted in Sweden in 2017 demonstrated that obesity is a risk factor for malaria [20].

Finally, we would like to emphasize the following key points and offer our recommendations for the management of surgical cases involving patients from malaria-endemic areas: (i) thorough questioning and examination for malaria should be conducted preoperatively; (2) implementing routine malaria screening tests may be beneficial for these patients; (iii) postoperative symptoms indicative of a malaria episode, such as fever, chills, and headache, necessitate prompt action for early diagnosis; (iv) diagnosis and treatment should be expedited without any delays; (v) establishing protocols for patients from malaria-endemic countries is advisable to prevent potential harm and fatalities associated with delayed suspicion, diagnosis, and treatment; (vi) protocols may vary based on the facilities and conditions of different countries and hospitals, but they contribute to establishing routines and increasing awareness in managing such cases.

## Conclusions

A thorough patient history and a high index of suspicion play a crucial role in diagnosing malaria infection. When evaluating postoperative fever, particularly in patients from malaria-endemic regions, it is essential to consider the possibility of malaria infection after eliminating common causes of fever. Additionally, surgical stress may potentially exacerbate the course of malaria infection.
